# Synbiotic Effects of Enzyme and Probiotics on Intestinal Health and Growth of Newly Weaned Pigs Challenged With Enterotoxigenic F18^+^ *Escherichia coli*

**DOI:** 10.3389/fvets.2020.00573

**Published:** 2020-09-09

**Authors:** Marcos Elias Duarte, James Tyus, Sung Woo Kim

**Affiliations:** ^1^Department of Animal Science, North Carolina State University, Raleigh, NC, United States; ^2^BioResource International, Inc., Durham, NC, United States

**Keywords:** *Escherichia coli*, growth performance, intestinal health, newly weaned pigs, probiotics, synbiotics, xylanase

## Abstract

This study aimed to investigate the effect of dietary supplementation with xylanase and probiotics on growth performance and intestinal health of nursery pigs challenged with enterotoxigenic *Escherichia coli* (ETEC). Sixty-four newly weaned pigs (32 barrows and 32 gilts with 7.9 ± 0.4 kg BW) were allotted in a randomized complete block design (2 × 2 factorial). Two factors were ETEC challenge (oral inoculation of saline solution or *E. coli* F18^+^ at 6 × 10^9^ CFU) and synbiotics (none or a combination of xylanase 10,000 XU/kg and *Bacillus* sp. 2 × 10^8^ CFU/kg). All pigs were fed experimental diets following NRC (2012) in two phases (P1 for 10 d and P2 for 11 d). The ETEC was orally inoculated on d 7 after weaning. Feed intake and BW were measured on d 7, 10, 15, and 20. On d 20, pigs were euthanized to collect samples to measure gut health parameters and microbiome. Synbiotics increased (*P* < 0.05) ADG in phase 1 and ETEC reduced (*P* < 0.05) ADG and G:F in the post-challenge period. ETEC increased (*P* < 0.05) the fecal score of pigs from d 7 to 13; however, synbiotics reduced (*P* < 0.05) it at d 9 and 11 in challenged pigs. ETEC increased (*P* < 0.05) mucosal MDA, IL-6, Ki-67^+^, and crypt depth, whereas synbiotics tended to reduce TNFα (*P* = 0.093), protein carbonyl (*P* = 0.065), and IL-6 (*P* = 0.064); reduced (*P* < 0.05) crypt depth and Ki-67^+^; and increased (*P* < 0.05) villus height. ETEC reduced (*P* < 0.05) the relative abundance of Bacteroidetes and Firmicutes and increased (*P* < 0.05) the relative abundance of Proteobacteria. In conclusion, ETEC challenge reduced growth performance by affecting microbiome, immune response, and oxidative stress in the jejunum. Synbiotics enhanced growth performance by reducing diarrhea, immune response, and oxidative stress in the jejunum.

## Introduction

Weaning is a challenging period for nursery pigs especially with their immune and intestinal functions resulting in reduced growth performance (1). During this period, pigs experience environmental, immunological, psychological, and nutritional challenges (2–4). Consequently, pigs at weaning are highly susceptible to pathogenic microorganism, such as enterotoxigenic *Escherichia coli* (ETEC) causing enteric diseases (5, 6). Moreover, the increasing pressure to ban the use of antibiotics as growth promoter (AGP) around the world due to the concern about microbial resistance has been a big challenge to the swine industry and researchers to maintain the gut health and performance of pigs (7–9).

Among these, newly weaned pigs have a low capacity to digest nutrients from plant-based feed because of the immaturity of the gastrointestinal tract (GIT), the different nutritional composition, compared with milk, and the anti-nutritional content that can damage the intestinal epithelial layers (10). Typical plant-based feeds contains ~2.3–3.8% of xylans, the main non-starch polysaccharides (NSP) (11, 12) increasing digesta viscosity, altering intestinal morphology, and reducing nutrient digestibility (13–16), which can induce a propitious environment for the growing of harmful bacteria, changing the gut associate microbiota in newly weaned pigs (17).

Nutritionally, numerous strategies have been attempted to eliminate or mitigate the effect of these challenges and produce healthy animals. Exogenous enzymes, such as xylanase, have been successfully used to hydrolyze the β-1,4 backbone of xylan, releasing xylan oligosaccharides (XOS) and, consequently, reducing the NSP content and the viscosity of digesta, increasing the digestibility of nutrients (14, 18, 19). Probiotics, such as bacteria from the genus *Bacillus*, are being largely used as an alternative to promote health and performance in the livestock industry. The genus *Bacillus* is well-known for its ability to form spores, produce antimicrobial compounds, and produce exogenous enzymes that are related to the ability to utilize different carbohydrate sources including those derived from plants, such as XOS (5, 20).

It is hypothesized that xylanase combined with *Bacillus* sp. as a synbiotic enhances growth performance of newly weaned pigs challenged with ETEC by enhancing the gut health and modulating the microbiome in the intestine by altering digesta viscosity. Thus, this study aimed to evaluate the supplemental effects of synbiotics on gut health and growth of newly weaned pigs challenged with ETEC.

## Materials and Methods

### Animals, Experimental Design, Additives, and Diets

The experimental procedures used in this study were reviewed and approved by the Institutional Animal Care and Use Committee at North Carolina State University following the North Carolina State Animal Care and Use Procedures (REG 10.10.01).

Sixty four newly weaned pigs (32 barrows and 32 gilts) at 21 d of age, with an initial body weight at 7.9 ± 0.4 kg, were allotted in a randomized complete block design in a 2 × 2 factorial arrangement, with ETEC challenge (oral inoculation of saline solution or *E. coli* F18^+^ at 6 × 10^9^ CFU) and synbiotics (none and a combination of xylanase 10,000 XU/kg and *Bacillus* sp. 2 × 10^8^ CFU/kg) as two factors. Pigs (PIC 337 × Camborough 22) were purchased from a commercial farm in North Carolina, USA. Sows and piglets used in this study were not vaccinated against *E. coli*. The symbiotic used in this study was EnzaPro obtained from BioResource International Inc. (Durham, NC). Each factor had 16 pens (*n*=16; eight pens with barrows and eight pens with gilts; and four body weight blocks within sex) and pigs were housed individually in a pen.

Pigs were fed the assigned experimental diets meeting the nutritional requirements suggested by NRC (21) for 20 d based on two phases (Phase 1: 10 d; and Phase 2: 10 d). The composition of mash basal diets is shown in Table 1.

**Table 1:** Composition of basal diets (as-fed basis).

**Ingredient, %**	**Phase 1**	**Phase 2**
Corn	35.46	36.77
Soybean meal	20.00	25.00
Corn DDGS	10.00	20.00
Whey permeate^a^	18.00	8.00
Poultry meal	5.00	2.00
Fish meal	4.00	0.00
Blood plasma	3.50	2.00
L-Lys HCl	0.48	0.45
DL-Met	0.18	0.11
L-Thr	0.13	0.08
Limestone	0.80	1.40
Vitamin premix^b^	0.03	0.03
Mineral premix^c^	0.15	0.15
Salt	0.22	0.22
Dicalcium phosphate	0.05	0.40
Poultry fat	2.00	3.40
**Calculated composition**		
ME, kcal/kg	3,436	3,437
Crude protein	24.83	24.05
SID^d^ Lys, %	1.50	1.35
SID Met + Cys, %	0.82	0.74
SID Trp, %	0.25	0.23
SID Thr, %	0.88	0.79
Ca, %	0.85	0.81
STTD^e^ P, %	0.45	0.40
**Analyzed composition**		
Dry matter, %	90.36	88.87
Crude protein, %	24.72	24.13
Neutral detergent fiber, %	7.82	9.71
Acid-detergent fiber, %	3.42	4.37
Crude fat, %	4.82	6.16
Ca, %	0.85	0.82
Total P, %	0.68	0.60

a *DairyLac80 (International Ingredient Corporation) was used as a source of whey permeate containing (79.3 ± 0.8) % lactose*.

b *The vitamin premix provided the following per kilogram of complete diet: 6,613.8 IU of vitamin A as vitamin A acetate, 992.0 IU of vitamin D3, 19.8 IU of vitamin E, 2.64 mg of vitamin K as menadione sodium bisulfate, 0.03 mg of vitamin B12, 4.63 mg of riboflavin, 18.52 mg of D-pantothenic acid as calcium pantothenate, 24.96 mg of niacin, and 0.07 mg of biotin*.

c *The trace mineral premix provided the following per kilogram of complete diet: 4.0 mg of Mn as manganous oxide, 165 mg of Fe as ferrous sulfate, 165 mg of Zn as zinc sulfate, 16.5 mg of Cu as copper sulfate, 0.30 mg of I as ethylenediamine di-hydroiodide, and 0.30 mg of Se as sodium selenite*.

d *SID, standardized ileal digestible*.

e *STTD P, standardized total tract digestible phosphorus*.

The quantitation of the xylanase (endo-1,4-β-D-xylanase) activity in feeds was performed using modified XylX6 assay (Megazyme, Wicklow, Ireland) as described by Mangan et al. (22). Xylanase enzymatic activity is calculated relative to a reference standard added to 50 mM Trisodium Citrate pH 6.0 buffer measured at A400. One unit of xylanase activity is defined as the amount of enzyme needed for the release of 1 nmol of reducing sugars per second from 0.5% xylan from Beechwood at 50°C in 50 mM Trisodium Citrate pH 6.0. The xylanase enzymatic activity is shown in Table 2. The microbial counting in feeds was conducted at the microbiology lab of BioResource International, Inc.

**Table 2:** Xylanase activity and microbial count in the feed.

**Treatment**	**Phase 1**	**Phase 2**
**Xylanase**, **×1,000 XU/kg of feed**		
No symbiotic	0.42	1.62
Synbiotic	13.37	14.49
***Bacillus*** **sp.**, **×10**^**8**^ **CFU/kg of feed**		
No synbiotic	0.00	0.00
Synbiotic	2.00	2.00

### Experimental Procedures and Sampling

The inoculum of *E. coli* F18^+^ was prepared to be nalidixic acid resistant and produce heat-stable toxins A (STa) and heat-stable toxins B (STb), using a strain originally resistant to nalidixic acid following our standard protocol as previously described by Cutler et al. (23). The final concentration was 6 × 10^9^ CFU/mL, orally inoculated and divided into two doses.

All pigs were fed the experimental diets for 7 d (pre-challenge period) until ETEC was orally inoculated to 32 pigs on d 7 of the study. The unchallenged group (32 pigs) received an oral administration of sterile physiological saline. The fecal score was recorded by the same trained person from the d 3 to d 7 of the study to analyze the effects of the synbiotic in the pre-challenge period and to confirm that pigs assigned to the challenge group are in normal fecal score before the *E. coli* F18^+^ inoculation. The fecal score was also recorded from d 8 to d 20 of the study to analyze the effects of ETEC infection (24, 25). The fecal score was recorded using a 1–5 scale: (1) very firm stool, (2) normal firm stool, (3) moderately loose stool, (4) loose, watery stool, and (5) very watery stool.

After 20 d of feeding, 48 pigs, 12 per treatment, were selected based on initial BW (the heaviest and the lightest pigs within sex were excluded of sampling) and euthanized by exsanguination after the penetration of a captive bolt to head in order to remove the gastrointestinal tract for sample collection. Digesta from mid-jejunum (3 m after the duodenojejunal junction) was collected into a 50-mL tube, placed on ice, and carried to the lab for viscosity measurement. Tissues from mid-jejunum were collected, rinsed with 0.9% saline solution, and placed into a 50-mL tube with 10% buffered formaldehyde to be used for histological evaluation to measure villus height, crypt depth, and the ratio of Ki-67 positive cells to total cells in the crypt, as an indicator of the enterocyte proliferation rate (10, 26).

Segments of mid-jejunum were longitudinally opened and scraped to collect mucosa. Two samples per pig were placed into 2-mL tubes and stored at −80°C, after snap-freezing in liquid nitrogen immediately after collection. Jejunal mucosa samples (500 mg) were suspended in 1 mL of phosphate-buffered saline (PBS) and homogenized on ice using a tissue homogenizer (Tissuemiser; ThermoFisher Scientific Inc., Waltham, MA, USA). After centrifugation at 14,000 × *g* for 3 min, the supernatant was divided into six sets and stored at −80°C until analysis to measure the concentration of total protein, Tumor necrosis factor-alpha (TNFα), interleukin 6 (IL-6), interleukin 8 (IL-8), protein carbonyl, and malondialdehyde (MDA).

### Sample Processing and Analyses

Immediately after collection, digesta from jejunum were divided into two tubes (15 mL) per pig and centrifuged at 1,000 × *g* at 4°C for 10 min. Then, 2 mL from each tube was centrifuged at 10,000 × *g* at 4°C for 10 min. The supernatant obtained was transferred to another 1.5-mL tube and kept on ice until measurement. The viscosity was measured using a viscometer (Brookfield Digital Viscometer, Model DV-II Version 2.0, Brookfield Engineering Laboratories Inc., Stoughton, MA), set at 25°C. The viscosity measurement was the average between 45.0/s and 22.5/s shear rates, and the viscosity values were recorded as viscosity in millipascal-seconds (mPa·s) (10, 14).

The concentrations of total protein, TNFα, IL-6, IL-8, MDA, and protein carbonyl were measured by colorimetric methods using commercially available kits according to the instructions of the manufacturers. The absorbance was read using a plate reader (Synergy HT, BioTek Instruments, Winooski, VT) and the Gen5 Data Analysis Software (BioTek Instruments). The concentration was calculated based on the standard curve created from the concentration and absorbance of the respective standard.

Total protein concentration in the mucosa of jejunum was measured using a BCA Protein Assay (23225#, ThermoFisher Scientific Inc. Rockford, IL) following Jang and Kim (26). Before analysis, the samples were diluted (1:60) in PBS to meet the working range for 20–2,000 μg/mL. The absorbance was measured at 562 nm. The total protein concentration was used to normalize the concentrations of TNFα, IL-6, IL-8, MDA, and protein carbonyl.

The concentration of TNFα in the mucosa of the jejunum was measured using the porcine ELISA Kit (PTA00; R&D System Inc. Minneapolis, MN) following Weaver and Kim (27). The working range was 23.4–1,500 pg/mL. Absorbance was read at 450 nm and 540 nm and the TNFα concentration was expressed as pg/mg protein. The concentration of IL-6 in the mucosa of jejunum was determined using the ELISA Kit (P6000B; R&D System Inc.) following Jang and Kim (26). The working range was 18.8–1,200 pg/mL. Absorbance was read at 450 nm and 540 nm, and the IL-6 concentration was expressed as pg/mg protein. The concentration of IL-8 in the mucosa of jejunum was determined using the ELISA Kit (P8000; R&D System Inc.). Before analysis, the samples were diluted (1:6) in PBS to meet the working range of 62.5–4,000 pg/mL. Absorbance was read at 450 and 540 nm, and the IL-8 concentration was expressed as ng/mg protein.

Malondialdehyde concentration in the mucosa of jejunum was measured using the Thiobarbituric Acid Reactive Substance Assay Kit (STA-330, Cell Biolabs, San Diego, CA) following Duarte et al. (10). The working range was 0.98–125 μmol/L. The absorbance was measured at 532 nm, and the MDA concentration mucosa was expressed as μmol/mg of protein.

Protein carbonyl concentration was measured using the ELISA kit (STA-310, Cell Biolabs) following Zhao and Kim (28). Before the analysis, the sample was diluted to reach a protein concentration of 10 μg/mL. The working range was 0.375–7.5 nmol/mL. The absorbance was read at 450 nm, and the protein carbonyl concentration was expressed as nmol/mg of protein.

Two sections of jejunum per pig fixed in 10% buffered formalin were sent to the North Carolina State University Histology Laboratory (College of Veterinary Medicine, Raleigh, NC). The sections were dehydrated, embedded in paraffin, and stained using hematoxylin and eosin dyes for morphological measurement and Ki-67 immunohistochemistry assay to detect Ki-67 positive cells according to Jang and Kim (26).

Villus height, villus width, and crypt depth were measured using a microscope Olympus CX31 (Lumenera Corporation, Ottawa, Canada) with a camera Infinity 2-2 digital CCD following Kim et al. (29). Lengths of ten well-oriented intact villi and their associated crypts were measured in each slide. The villi length was measured from the top of the villi to the villi-crypt junction, the villi width was measured in the middle of the villi, and the crypt depth was measured from the villi-crypt junction to the bottom of the crypt. Then, the villus height to crypt depth (VH:CD) ratio was calculated. Images of 10 intact crypts from each slide were cropped, and the ImageJS software was used for calculating the percentage of Ki-67 positive cells to total cells in the crypt. All analyses of the intestinal morphology were executed by the same person. The averages of the 10 measurements per pig were calculated and reported as one number per pig.

Jejunal mucosa samples were used to extract DNA content to analyze the microbiome. The DNA extraction was performed using the DNA Stool Mini Kit (#51604, Qiagen; Germantown, Maryland, USA) and following the instructions of the manufacturer. Samples were sent to Mako Medical Laboratories (Raleigh, NC, USA) for microbial sequencing using the 16S rRNA technique following Kim et al. (29). Briefly, the Ion Chef instrument was used to prepare the samples for template and sequencing was performed on the Ion S5 system (ThermoFisher, Inc., Wilmington, DE, USA). Variable regions V2, V3, V4, V6, V7, V8, and V9 of the 16S rRNA gene were amplified with the Ion 16S Metagenomics Kit 113 (ThermoFisher Scientific). Sequences were processed using the Torrent Suite Software (version 5.2.2) (ThermoFisher Scientific) to produce raw unaligned sequence data files for further analysis. Sequence data analysis, alignment to GreenGenes and MicroSeq databases, alpha and beta diversity plot generation, and OTU table generation were performed by the Ion Reporter Software Suite (version 5.2.2) of bioinformatics analysis tools (ThermoFisher Scientific). Samples were analyzed using Ion Reporter's Metagenomics 16S workflow powered by Qiime (version w1.1). The OTU data were transformed to relative abundance before statistical analysis. The OTU data with the relative abundance <0.05% within each level were combined as “Others”.

### Statistical Analyses

Pigs were allotted in a randomized complete block design using sex and the initial BW as blocks in all measurements to account for the variation of the initial BW and sex dimorphism (30). The experimental unit was the pig, individually housed and fed. The main effects were the factors (ETEC challenge and synbiotics) and their interaction. Factors were handled as fixed effects, and initial BW and sex blocks were handled as random effects. For growth performance and fecal score data, each factor had 16 pigs (*n* = 16; eight barrows and eight gilts; and four body weight blocks within sex). For other data, each factor had 12 pigs (*n*=12; six barrows and eight gilts; and three body weight blocks within sex). Data were analyzed using the Mixed procedure in SAS version 9.4 (SAS Inc., Cary, NC, USA). The means were separated using the LSMEANS statement in SAS. When an interaction between the factors was significant or tended to be significant, a pairwise comparison was made using the PDIFF option in SAS. Statistical differences were considered significant with *P* < 0.05. Tendency was considered when 0.05 ≤ *P* < 0.10.

## Results

### Growth Performance

In the pre-challenge period, the synbiotic tended to increase (*P* = 0.059) the BW of pigs at d 7 after weaning (Table 3). At d 10 of the study, 3 days post-challenge, the oral inoculation of ETEC did not affect the BW, whereas it was increased (*P* < 0.05) by the dietary use of synbiotics. However, at d 20 of the study, the BW of pigs challenged with ETEC was reduced (*P* < 0.05), whereas it was not affected by the synbiotic.

**Table 3:** Growth performance of pigs challenged with ETEC (CH) on d 7 post-weaning and fed diets supplemented with a synbiotic (SY).

**Challenged synbiotic**	**–^1^**		**+^1^**		**SEM**	***P-*****value**		
	**–^2^**	**+^2^**	**–^2^**	**+^2^**		**CH**	**SY**	**CH × SY**
**BW, kg**								
Initial	7.91	7.92	7.90	7.92	0.39	0.929	0.829	0.956
d 7	8.02	8.37	8.09	8.23	0.36	0.776	0.059	0.401
d 10	8.57	9.04	8.31	8.73	0.38	0.122	0.020	0.885
d 20	13.04	13.42	12.08	12.62	0.67	0.022	0.227	0.818
**ADG, g/d**								
Pre-challenge	15	65	27	44	20	0.768	0.032	0.284
Post-challenge	387	388	307	338	29	0.013	0.517	0.547
Phase 1	66	112	41	80	18	0.092	0.014	0.839
Phase 2	447	438	377	390	33	0.023	0.934	0.652
Overall	257	275	209	235	19	0.019	0.220	0.812
**ADFI, g/d**								
Pre-challenge	111	140	147	142	15	0.177	0.402	0.235
Post-challenge	459	498	437	466	29	0.289	0.188	0.847
Phase 1	156	199	176	195	16	0.606	0.054	0.457
Phase 2	519	547	494	523	36	0.358	0.281	0.989
Overall	337	373	335	353	21	0.571	0.173	0.638
**G:F**								
Pre-challenge	0.16	0.47	0.19	0.30	0.12	0.531	0.066	0.370
Post-challenge	0.84	0.78	0.70	0.71	0.04	0.008	0.500	0.385
Phase 1	0.51	0.57	0.29	0.44	0.06	0.010	0.098	0.478
Phase 2	0.87	0.80	0.76	0.73	0.03	0.004	0.126	0.617
Overall	0.76	0.74	0.62	0.66	0.03	0.003	0.811	0.437

1 *ETEC challenge*.

2 *Synbiotic*.

At the pre-challenge period (d 0–7) the synbiotic increased (*P* < 0.05) the ADG of pigs. On the post-challenge period (d 7–20), the ADG of pigs challenged with ETEC was reduced (*P* < 0.05). In phase 1 (d 0–10), the ETEC challenge tended to reduce (*P* = 0.092) the ADG, whereas the use of synbiotics increased (*P* < 0.05) it, regardless of the challenge. In phase 2 (d 10–20) and overall, the ADG was reduced (*P* < 0.05) when pigs were challenged with ETEC, whereas it was not affected by the use of synbiotics.

The ADFI was not affected by the use of synbiotics during the pre-challenge period (d 0–7). In the post-challenge period, the ETEC challenge did not affect the ADFI, whereas, the use of synbiotics tended to increase (*P* = 0.054) the ADFI during phase 1 (d 0–10). The ADFI was not affected by the two factors during phase 2 and overall.

At the pre-challenge period (d 0–7) the use of synbiotics tended to increase (*P* = 0.066) the G:F ratio of pigs. However, in the post-challenge period (d 7–20), the G:F ratio of pigs challenged with ETEC was reduced (*P* < 0.05). At phase 1, the G:F ratio was reduced (*P* < 0.05) when pigs were challenged with ETEC, whereas it tended to increase (*P* = 0.098) by the use of synbiotics. In phase 2 and overall, the G:F ratio was reduced (*P* < 0.05) when pigs were challenged with ETEC, whereas it was not affected by the use of synbiotics.

### Fecal Score

**Figure 1:**
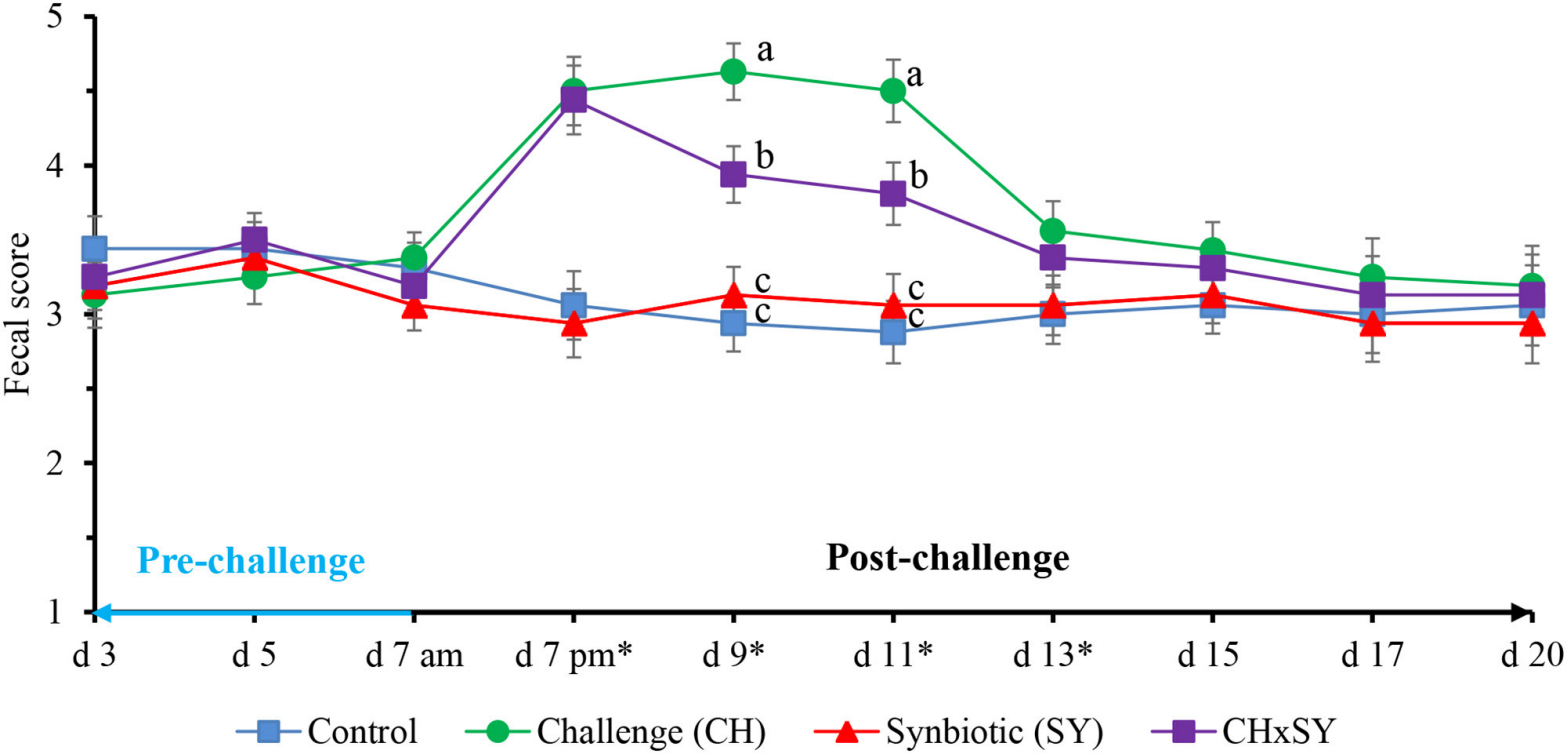
Fecal score of pigs challenged with ETEC (CH) on d 7 post-weaning and fed diets supplemented with a synbiotic (SY). * **d 7 pm**: CH: (*P* < 0.001), SY: (*P* = 0.685), CH × SY: (0.892); **d 9**: CH: (*P* < 0.001), SY: (*P* = 0.124), CH × SY: (*P* < 0.05); **d 11**: CH: (*P* < 0.001), SY: (*P* = 0.236), CH × SY: (*P* < 0.05); **d 13**: CH: (*P* < 0.05), SY: (*P* = 0.718), CH × SY: (*P* = 0.471). ^a,b^ Within a column, means without a common superscript letter differ (*P* < 0.05).

The fecal score of pigs challenged with ETEC was increased (*P* < 0.05) at d 7, 9, 11, and 13; however, at d 9 and 11, the use of synbiotics reduced (*P* < 0.05) the fecal score of pigs challenged with ETEC (Figure 1). The fecal score was not affected by the two factors at d 15, 17, and 20.

### Immune and Oxidative Status

The mucosal concentration of MDA was increased (*P* < 0.05) when pigs were challenged with ETEC, whereas it was not affected by the use of synbiotics (Table 4). However, the concentration of protein carbonyl was not affected when pigs were challenged with ETEC, whereas it tended to reduce (*P* = 0.065) with the use of synbiotics. The concentration of TNFα was not affected when pigs were challenged with ETEC, whereas, it tended to reduce (*P* = 0.093) with the use of synbiotics. The concentration of IL-6 was increased (*P* < 0.05) when pigs were challenged with ETEC, whereas it tended to decrease (*P* = 0.064) with the use of synbiotics, regardless of the challenge. The concentration of IL-8 was not affected by the factors.

**Table 4:** Oxidative stress and immune parameters in the jejunal mucosa of pigs challenged with ETEC (CH) on d 7 post-weaning and fed diets supplemented with a synbiotic (SY).

**Challenged synbiotic**	**–^1^**		**+^1^**		**SEM**	***P-*****value**		
	**–^2^**	**+^2^**	**–^2^**	**+^2^**		**CH**	**SY**	**CH × SY**
MDA, μmol/mg of protein	0.24	0.28	0.88	0.76	0.10	<0.001	0.713	0.412
Protein carbonyl, nmol/mg of protein	3.02	2.41	3.24	2.60	0.38	0.529	0.065	0.957
TNFα, pg/mg of protein	0.97	0.86	1.13	0.89	0.11	0.439	0.093	0.645
IL-8, ng/mg of protein	0.49	0.52	0.51	0.53	0.05	0.825	0.546	0.924
IL-6, pg/mg of protein	3.16	2.76	4.98	3.62	0.46	0.006	0.064	0.306

1 *ETEC challenge*.

2 *Synbiotic*.

### Histomorphology, Immunohistochemistry, and Digesta Viscosity

The enterotoxigenic *E. coli* challenge at d 7 post-weaning reduced (*P* < 0.05) the villus height and VH:CD ratio and increased (*P* < 0.05) the crypt depth and the ratio of Ki-67 positive cells to total cell in the crypt in jejunum of pigs (Table 5), whereas, regardless of the ETEC challenge, the use of synbiotics increased (*P* < 0.05) the villus height and VH:CD ratio, reduced (*P* < 0.05) the crypt depth, and tended to reduce (*P* = 0.053) the ratio of Ki-67 positive cells to total cell in the crypt in the jejunum of pigs. The viscosity of the jejunal digesta was not affected by the factors.

**Table 5:** Jejunal histomorphology and digesta viscosity of pigs challenged with ETEC (CH) on d 7 post-weaning and fed diets supplemented with a synbiotic (SY).

**Challenged synbiotic**	**–^1^**		**+^1^**		**SEM**	***P-*****value**		
	**–^2^**	**+^2^**	**–^2^**	**+^2^**		**CH**	**SY**	**CH × SY**
Villus height, μm	405.3	434.1	331.6	408.6	25.5	0.005	0.003	0.153
Villus width, μm	99.7	90.6	95.4	91.4	4.7	0.698	0.153	0.569
Crypt depth, μm	273.6	246.5	306.2	273.9	10.7	0.002	0.003	0.782
VH:CD ratio^3^	1.50	1.80	1.11	1.52	0.09	0.001	0.001	0.534
Ki-67 positive, %^4^	29.95	28.25	35.46	32.34	1.21	0.001	0.053	0.559
Viscosity, mPa·s	1.92	1.90	1.88	1.76	0.07	0.193	0.354	0.465

1 *ETEC challenge*.

2 *Synbiotic*.

3 *Villus height to crypt depth ratio*.

4 *The ratio of Ki-67 positive cells to total cells in the crypt*.

### Microbiome

**Figure 2:**
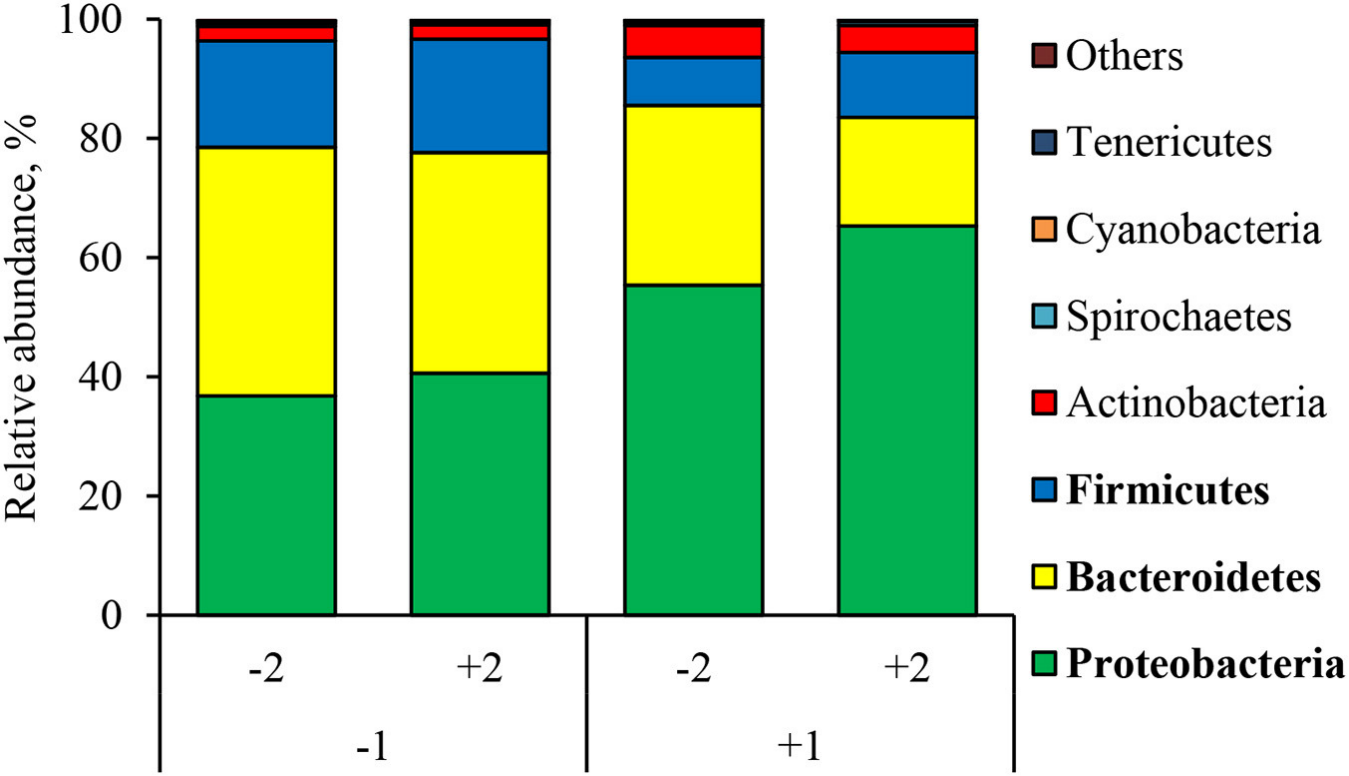
Relative abundance of jejunal mucosa-associated microbiota at the phylum level in pigs challenged with ETEC (CH) on d 7 post-weaning and fed diets supplemented with a synbiotic (SY). Each pattern represents a particular bacterial phylum. Phylum sequences that did not achieve 0.5% within each phylum were combined as “Others.” 1: ETEC challenge (CH); 2: Synbiotic (SY). **Proteobacteria**: CH: (*P* < 0.05), SY: (*P* = 0.339), CH × SY: (*P* = 0.668). **Bacteroidetes**: CH: (*P* < 0.05), SY: (*P* = 0.162), CH × SY: (*P* = 0.542); **Firmicutes**: CH: (*P* < 0.05), SY: (*P* = 0.523), CH × SY: (*P* = 0.803).

At the phylum level (Figure 2), the ETEC reduced (*P* < 0.05) the relative abundance of Bacteroidetes and Firmicute and increased (*P* < 0.05) the relative abundance of Proteobacteria. The synbiotic did not affect the relative abundance of microbials at the phylum level. At the family level (Table 6), pigs challenged with ETEC tended to reduce the relative abundance of *Clostridiaceae* (*P* = 0.067) and *Prevotellaceae* (*P* = 0.069), and reduced (*P* < 0.05) the relative abundance of *Veillonellaceae*, whereas it tended to increase (*P* = 0.063) the relative abundance of *Helicobacteraceae*. The use of synbiotics did not affect the jejunal mucosa-associated microbiota at the family level. In the genus level (Table 7), the ETEC challenge reduced (*P* < 0.05) the relative abundance of *Megasphaera, Mitsuokella*, and *Selenomonas* and tended to reduce (*P* = 0.060) the relative abundance of *Helicobacter*, whereas the use of synbiotics did not affect the jejunal mucosa-associated microbiota at the genus level. At the species level (Table 8), the ETEC challenge reduced (*P* < 0.05) the relative abundance of *Acidaminococcus fermentans, Selenomonas bovis*, and *Selenomonas lipolytica* and tended to decrease the relative abundance of *Prevotella copri* (*P* = 0.096) and *Roseburia faecis* (*P* = 0.079). Pigs fed synbiotics increased (*P* < 0.05) the relative abundance of *Helicobacter_mastomyrinus* in unchallenged pigs compared with the control group. Pigs challenged with ETEC and fed a diet with synbiotics increased (*P* < 0.05) the relative abundance of *Campylobacter coli* compared with pigs fed synbiotics and not challenged. Pigs challenged with ETEC and fed a diet with a synbiotic tended to increase (*P* = 0.075) the relative abundance of *Campylobacter hyointestinalis* compared with pigs fed synbiotics and not challenged.

**Table 6:** Relative abundance of jejunal mucosa-associated microbiota at the family level in pigs challenged with ETEC (CH) on d 7 post-weaning and fed diets supplemented with a synbiotic (SY).

**Challenged synbiotic**	**–^1^**		**+^1^**		**SEM**	***P-*****value**		
	**–^2^**	**+^2^**	**–^2^**	**+^2^**		**CH**	**SY**	**CH × SY**
*Helicobacteraceae*	30.13	38.9	43.55	55.41	14.35	0.063	0.196	0.845
*Prevotellaceae*	42.16	33.89	32.48	21.88	11.52	0.069	0.111	0.841
*Lactobacillaceae*	4.32	6.70	4.81	5.35	2.07	0.836	0.485	0.661
*Veillonellaceae*	8.07	6.15	3.19	2.84	1.34	0.003	0.380	0.545
*Corynebacteriaceae*	2.16	2.24	4.43	4.07	2.01	0.313	0.945	0.913
*Campylobacteraceae*	2.42	1.46	1.66	1.22	0.69	0.461	0.302	0.698
*Lachnospiraceae*	1.40	1.60	0.96	1.45	0.40	0.418	0.348	0.695
*Succinivibrionaceae*	0.77	0.28	1.88	1.24	0.96	0.159	0.444	0.920
*Clostridiaceae*	1.38	0.82	0.51	0.58	0.30	0.067	0.412	0.294
*Ruminococcaceae*	0.94	0.95	0.56	0.66	0.33	0.192	0.830	0.859
*Eubacteriaceae*	0.74	0.78	0.70	0.88	0.33	0.856	0.559	0.705
*Porphyromonadaceae*	0.74	0.76	0.52	0.67	0.24	0.417	0.665	0.735
*Enterobacteriaceae*	0.27	1.13	0.32	0.52	0.45	0.522	0.229	0.449
*Bacillaceae*	0.04	0.13	0.05	0.04	0.05	0.420	0.419	0.357
Others	4.47	4.27	4.38	4.35	1.12	0.999	0.904	0.928

1 *ETEC challenge*.

2 *Synbiotic*.

**Table 7:** Relative abundance of jejunal mucosa-associated microbiota at the genus level in pigs challenged with ETEC (CH) on d 7 post-weaning and fed diets supplemented with a synbiotic (SY).

**Challenged synbiotic**	**–^1^**		**+^1^**		**SEM**	***P-*****value**		
	**–^2^**	**+^2^**	**–^2^**	**+^2^**		**CH**	**SY**	**CH × SY**
*Helicobacter*	33.34	42.25	49.15	57.52	13.68	0.060	0.289	0.973
*Prevotella*	42.42	34.70	31.01	24.16	12.05	0.117	0.294	0.949
*Lactobacillus*	5.77	8.02	5.60	6.04	2.66	0.688	0.616	0.735
*Corynebacterium*	2.79	2.79	4.89	4.42	2.18	0.398	0.915	0.913
*Campylobacter*	3.16	1.80	1.96	1.49	0.86	0.367	0.275	0.591
*Mitsuokella*	2.60	1.19	0.83	0.66	0.68	0.041	0.153	0.27
*Selenomonas*	1.87	2.39	0.55	0.24	0.52	<0.001	0.818	0.377
*Succinivibrio*	0.85	0.22	1.72	1.22	0.93	0.208	0.446	0.928
*Megasphaera*	1.09	0.83	0.35	0.24	0.21	0.002	0.366	0.713
Others	5.30	4.99	3.46	3.82	0.96	0.124	0.975	0.727

1 *ETEC challenge*.

2 *Synbiotic*.

**Table 8:** Relative abundance of jejunal mucosa-associated microbiota at the species level in pigs challenged (CH) with ETEC on d 7 post-weaning and fed diets supplemented with synbiotics (SY).

**Challenged synbiotic**	**–^1^**		**+^1^**		**SEM**	***P-*****value**		
	**–^2^**	**+^2^**	**–^2^**	**+^2^**		**CH**	**SY**	**CH × SY**
*Prevotella copri*	39.80	30.59	29.55	20.72	7.94	0.096	0.135	0.974
*Helicobacter rappini*	16.12	23.06	26.76	27.82	6.97	0.228	0.529	0.643
*Helicobacter mastomyrinus*	4.12^b^	13.80^a^	10.00^ab^	8.05^ab^	3.47	0.983	0.193	0.053
*Prevotella stercorea*	7.39	7.65	6.13	8.42	3.65	0.913	0.561	0.642
*Corynebacterium glutamicum*	3.61	2.22	4.08	4.60	2.11	0.504	0.838	0.653
*Helicobacter equorum*	0.07	0.45	2.54	10.07	4.40	0.107	0.287	0.336
*Lactobacillus mucosae*	2.44	4.18	0.99	2.52	1.51	0.309	0.285	0.942
*Prevotella sp*.	2.30	1.85	3.59	1.97	1.95	0.648	0.507	0.703
*Corynebacterium deserti*	2.11	1.17	2.26	2.20	1.15	0.611	0.662	0.705
*Lactobacillus kitasatonis*	1.18	1.05	1.70	1.85	0.72	0.363	0.984	0.843
*Campylobacter upsaliensis*	2.31	0.87	1.45	0.33	1.05	0.506	0.230	0.883
*Mitsuokella jalaludinii*	1.47	0.81	0.86	0.69	0.53	0.360	0.297	0.548
*Lactobacillus delbrueckii*	1.12	1.01	0.40	0.68	0.43	0.225	0.838	0.653
*Selenomonas bovis*	1.08	1.50	0.33	0.24	0.29	0.001	0.582	0.384
*Dialister succinatiphilus*	0.93	0.79	0.63	0.48	0.30	0.247	0.593	0.989
*Roseburia faecis*	0.90	0.83	0.47	0.54	0.24	0.079	0.986	0.724
*Lactobacillus sp*.	0.86	0.66	0.35	0.67	0.31	0.430	0.837	0.398
*Helicobacter canadensis*	1.00	0.13	0.30	1.08	0.70	0.842	0.943	0.202
*Prevotella ruminicola*	0.64	0.18	1.03	0.27	0.52	0.673	0.296	0.799
*Selenomonas lipolytica*	0.74	1.00	0.20	0.15	0.22	0.004	0.650	0.491
*Mitsuokella multacida*	1.12	0.29	0.30	0.37	0.48	0.378	0.361	0.280
*Faecalibacterium prausnitzii*	0.65	0.62	0.26	0.47	0.16	0.113	0.612	0.464
*Succinivibrio dextrinosolvens*	0.63	0.25	0.88	0.24	0.48	0.782	0.250	0.774
*Campylobacter coli*	0.57^AB^	0.19^B^	0.28^AB^	0.63^A^	0.17	0.637	0.960	0.031
*Phascolarctobacterium succinatutens*	0.22	0.32	0.27	0.67	0.27	0.243	0.148	0.394
*Acidaminococcus fermentans*	0.50	0.37	0.16	0.20	0.10	0.036	0.712	0.436
*Campylobacter lanienae*	0.34	0.15	0.23	0.40	0.11	0.507	0.909	0.113
*Lactobacillus amylovorus*	0.09	0.17	0.59	0.23	0.21	0.200	0.523	0.319
*Campylobacter hyointestinalis*	0.30^A^	0.14^B^	0.19^AB^	0.27^AB^	0.07	0.932	0.568	0.055
*Pelomonas puraquae*	0.10	0.21	0.16	0.31	0.09	0.375	0.160	0.804
Others	4.25	3.76	3.19	3.27	0.73	0.290	0.782	0.699

1 *ETEC challenge*.

2 *Synbiotic*.

a, b *Within a row, means without a common superscript letter differ (P < 0.05)*.

A, B *Within a row, means without a common superscript letter differ (P < 0.10)*.

**Figure 3:**
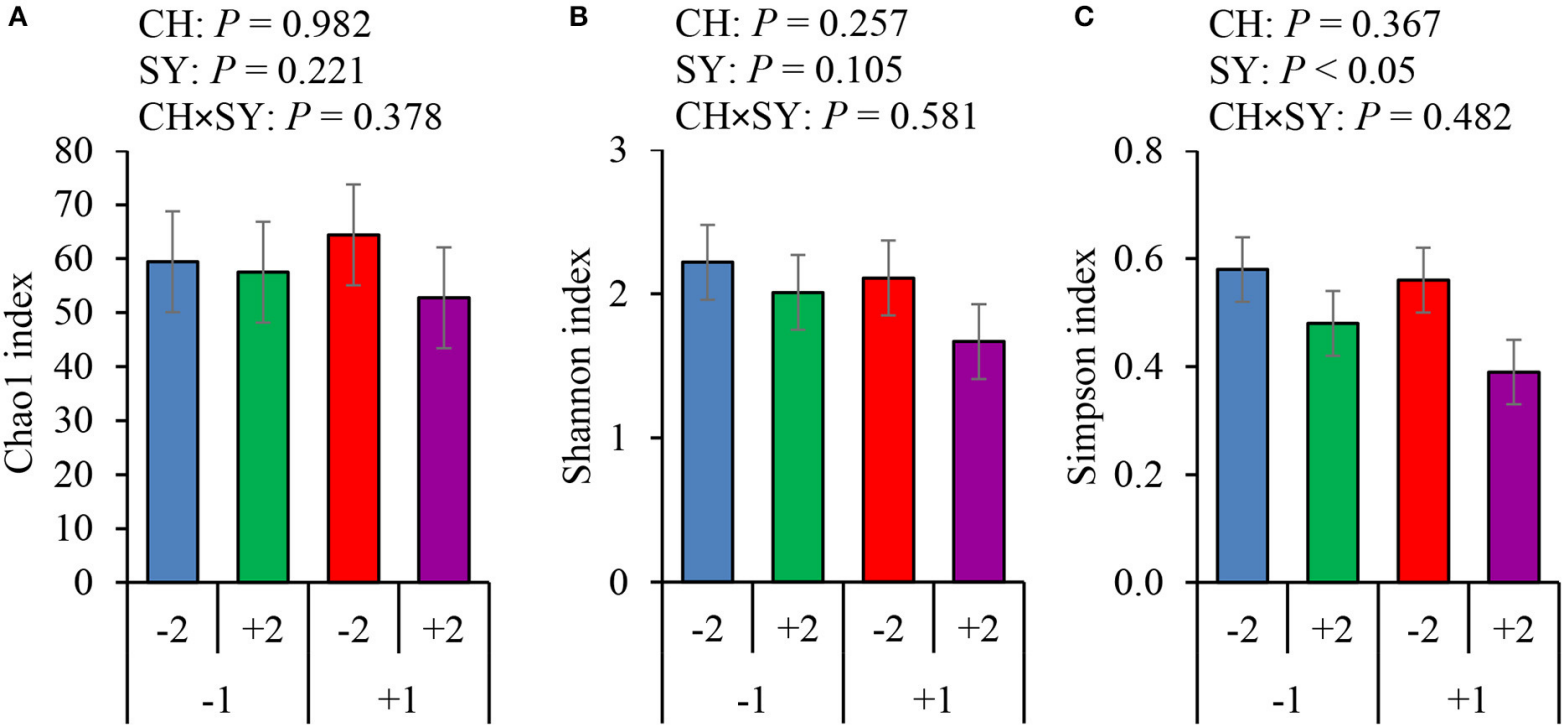
Alpha diversity of jejunal mucosa-associated microbiota at the family level estimated with Chao1 richness **(A)**, Shannon diversity **(B)**, and Simpson diversity **(C)** in pigs challenged (CH) with ETEC on d 7 post-weaned and fed diets supplemented with a synbiotic (SY). 1: ETEC challenge; 2: Synbiotic; SY: synbiotic; CH: challenge; CH × SY: Challenge and synbiotic.

**Figure 4:**
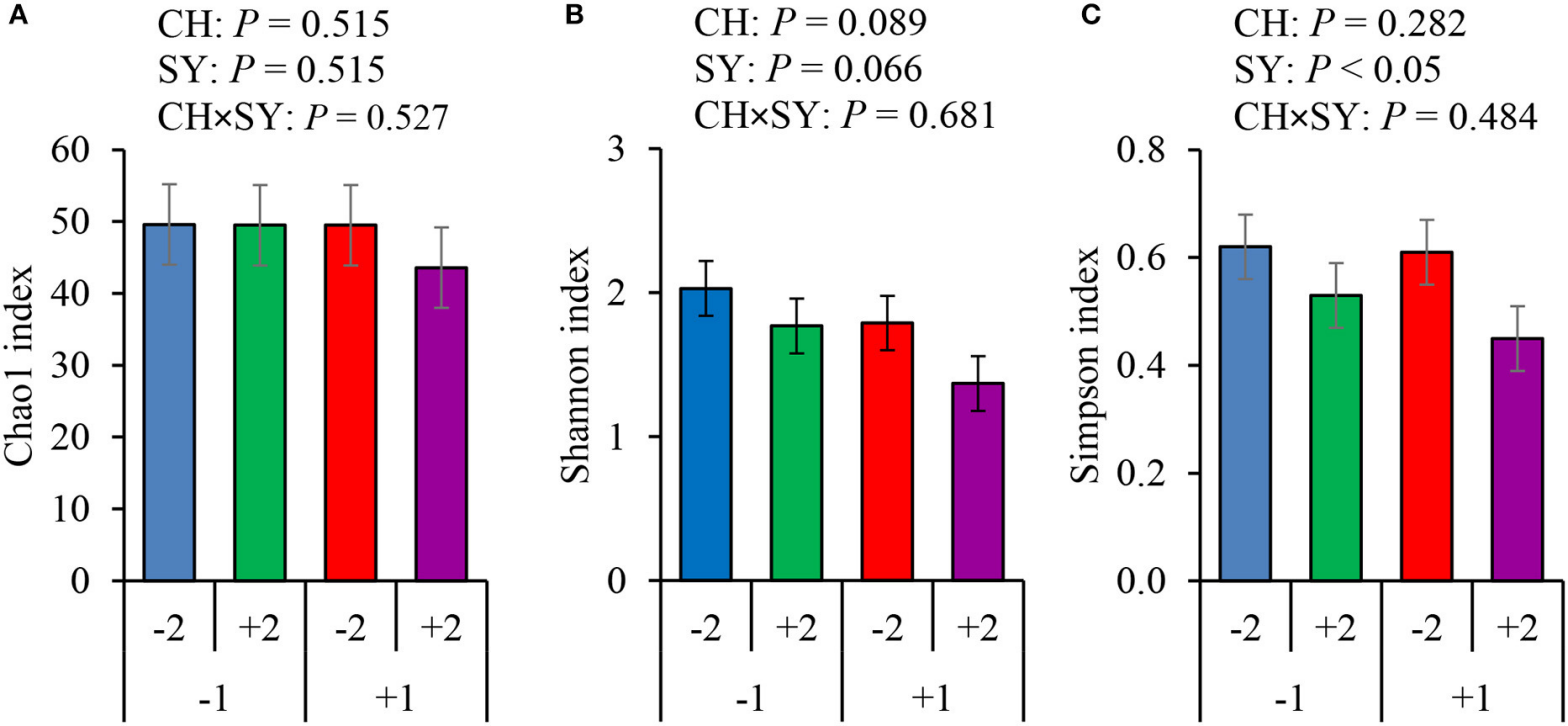
Alpha diversity of jejunal mucosa-associated microbiota at the genus level estimated with Chao1 richness **(A)**, Shannon diversity **(B)**, and Simpson diversity **(C)** in pigs challenged (CH) with ETEC on d 7 post-weaned and fed diets supplemented with a synbiotic (SY). 1: ETEC challenge; 2: Synbiotic; SY: synbiotic; CH: challenge; CH × SY: Challenge and synbiotics.

There was no effect of the factors on alpha diversity of jejunal mucosa-associated microbiota in pigs estimated with Chao1 richness estimator at the family (Figure 3A) and genus levels (Figure 4A). At the family level, the Shannon diversity index was not affected by the factors (Figure 3B), whereas at the genus level, it tended to be reduced by the ETEC challenge (*P* = 0.089) and the synbiotic (*P* = 0.066), regardless of the challenge (Figure 4B). The Simpson diversity index was not affected by the ETEC challenge, whereas it was reduced (*P* < 0.05) by the synbiotic at the family (Figure 3C) and genus levels (Figure 4C).

## Discussion

In this study, pigs were housed individually in order to know the intake of synbiotics affecting intestinal health following procedures previous described (26, 31, 32). The beneficial effects of the synbiotic shown in this study were prominent especially during the period immediately after the weaning when pigs receive the greatest nutritional challenges from plant-based diets, whereas the synbiotic seems to be efficient in enhancing the jejunal histomorphology and reducing the fecal score and the microbial diversity without affecting the growth performance during P2 of this study. Probiotics and prebiotics are shown to be effective to newly weaned pigs because of the immaturity of the intestine and limited digestive capacity of plant-based diets (10, 33). As pigs adapt to plant-based diets, however, pigs develop the intestine to handle fiber and utilize dietary nutrients more efficiently (34–36).

This study confirmed that *E. coli* F18^+^ can be associated with post-weaning diarrhea (PWD), reducing the growth, modulating the microbiome, and affecting the gut heath of newly weaned pigs as previously reported (5, 25, 37). Enterotoxins (including STa, and STb) from ETEC are a major cause of increased fecal score as shown in this study. The fimbria of the *E. coli* bind to glycoproteins in the microvilli of the intestine of newly weaned pigs by a fimbria receptor interaction causing an interference in the electrolytes fluid that leads to diarrhea by enterotoxin interaction (5, 38–40). The predisposition of newly weaned pigs to PWD caused by ETEC have been related to the psychological, environmental, and physiological stress after weaning, as well as sudden transition from sow's milk to plant-based diets that are solid and less digestible. These stressors disrupt the immune system and the intestinal microbiota leading to intestinal inflammation and PWD (41, 42), consequently reducing growth performance (5, 6, 17). As previously reported (31, 43), the challenge with *E. coli* F18^+^ in this study reduced growth and feed efficiency without affecting feed intake, which is in agreement with previous studies. Reduced feed efficiency in pigs with *E. coli* infection is related to impaired nutrient absorption (43, 44) and the activation of immune system partitioning nutrients from growth (45).

McLamb et al. (24) previously reported that pigs challenged with ETEC have the immune response activated. According to Loos et al. (46), the secretion of IL-6 in the lumen of the small intestine is stimulated by STa produced by *E. coli* F18^+^. In this study, the ETEC challenge increased the concentration of IL-6 as previous reported (46, 47). High levels of IL-6 reduce the secretion of growth hormone (48) and damage the intestinal epithelium (49, 50). The challenge, however, did not affect the concentration of IL-8 and TNFα in this study. According to Loos et al. (51), the IL-8 has low expression in response to ETEC. The activation of the immune system in response to the ETEC infection may lead to an exhaustion of the antioxidant mechanism causing the oxidation of cellular protein, lipids, and DNA (52). The results of this study show, on challenged pigs, an increasing level of MDA, a final product of the lipid oxidation, and an indicator of oxidative stress (53, 54). The metabolites from oxidative stress can directly affect the enterocytes' cell wall components, such as lipids and proteins, causing apoptosis and, consequently, reduction of villi length (54, 55). The villi reduction in the challenged pigs leads to increasing the crypt cell proliferation rate, and consequently, increasing the crypt depth which is in accordance with previous studies (3, 43, 56). The increased oxidative stress due to the activated immune system may also redirect energy and nutrients from growth to immune response, and to repair the epithelium.

The use of synbiotics, however, was effective to reduce the jejunal mucosal levels of IL-6, TNFα, and protein carbonyl regardless of the ETEC challenge. The reduction of the immune and oxidative stress indicators reduces the epithelial damage and the cell proliferation rate by reducing the deleterious effect of the ETEC on nursery pigs (43, 57–59). These results showed a potential benefit of dietary xylanase and *Bacillus* sp. as a synbiotic on enhancing the gut health and further reducing the jejunal mucosal protein carbonyl concentration. Therefore, this study targeted the investigation of the combinational effects of xylanase and *Bacillus* sp. The synbiotic had beneficial outcomes because xylanase successfully hydrolyzed xylans to XOS in feeds (10, 31, 60, 61), reducing the viscosity of digesta (10, 19) releasing nutrients for digestion (14, 18). Passos et al. (14) reported that dietary supplementation with xylanase showed a linear increase in the ileal digestibility of NDF, indicating the hydrolysis of NSP-releasing oligosaccharides, such as XOS. In addition, *Bacillus* sp. effectively utilizes XOS released by xylanase hydrolysis, further exerting synergetic effects (20) including their antibacterial properties (43, 62, 63).

The synbiotic can selectively affect the growth of microorganisms in the intestine, including those directly added in the diet (64, 65), targeting some metabolic processes and possibly changing the physical-chemical properties of the digesta (66). This mechanism can promote gut health benefits, such as modulation of gut microbiota by competition and antimicrobial property (67), and consequently, affect the immune system, reduce the oxidative stress, and increase the growth performance of newly weaned pigs (31).

Pigs from the challenge group-fed diets with the synbiotic had reduced diarrhea occurrence earlier than those without synbiotic supplementation. This outcome shows that the dietary supplementation of synbiotics may prevent ETEC from damaging the intestinal epithelium. Although the jejunal digesta viscosity was not affected by either factors in this study, the viscosity can be affected by the ingredient in the diet (19, 31) and the ratio of insoluble to soluble NSP (68). The viscosity observed in this study ranged from 1.8 to 1.9 mPa·s, which is lower than previously reported by Duarte et al. (10) and Passos et al. (14) due to differences in dietary compositions.

The ETEC slightly reduced the microbial diversity index but caused an imbalance in jejunal mucosa-associated microbiota by increasing the relative abundance of Proteobacteria by increasing the family *Helicobacteraceae* and the genus *Helicobacter*, consequently reducing the relative abundance of Bacteroidetes and Firmicutes, *Prevotellaceae*, and *Mitsukella and Selenomonas*, as previously reported by Bin et al. (69) and Pollock et al. (70). The adherence of the ETEC and the production of enterotoxins with the subsequent secretion of fluid to the intestinal lumen (41, 51) create a propitious environment to the growth of proteobacteria (17). The high abundance of *Helicobacteraceae* which belong to the Proteobacteria has been reported to cause a reduction of the mucous layer protection (65), which explains the impact of the challenge on the villus height, immune response, and the oxidative stress status, whereas, *Prevotellaceae*, which belongs to the Bacteroidetes has been related to intestinal mucosa of healthy pigs fed plant-based diets (71, 72).

The synbiotic reduced the diversity of the microbiome without affecting the relative abundance of microbials. *Bacillus* spores, *Lactobacillus acidophilus* and *Pediococcus acidilactici* used as probiotics has been reported to reduce the microbial diversity in pigs (33, 73). According to Poulsen et al. (33), *Bacillus* spores are able to adhere to intestinal epithelium and competitively affect the colonization pattern. Reduction on mucosa-associated microbial diversity have been related to increased inflammatory response (74), even though this was not observed in this study. These results may suggest that the type of the dominant microbials in the jejunal mucosa is more important to affect the intestinal immune response than the microbial diversity. According to Wang et al. (73), the ability of probiotics to reduce the diversity or richness of microbiome can positively affect the growth performance by reducing the deleterious effects of harmful microbes that can affect the immune system, oxidative stress, and intestinal histomorphology. It was confirmed in this study that the synbiotic supplementation increased growth performance, and villus height, reducing diarrhea, immune response, and oxidative stress in nursery pigs.

In conclusion, the ETEC challenge reduced the growth performance of newly weaned pigs by increasing the relative abundance of harmful bacteria, intestinal immune response, intestinal oxidative stress, and crypt depth while reducing the villus height in the small intestine. Dietary supplementation of xylanase and *Bacillus* sp. as a synbiotic enhanced growth performance by increasing the relative abundance of beneficial bacteria in the small intestine, reducing diarrhea, reducing the oxidative stress, and increasing the villus height in the small intestine regardless of the challenge. The synbiotic showed potential benefits on growth performance, reducing diarrhea, immune response, and the oxidative stress status in the small intestine, leading to a protective function on the intestinal epithelium. Therefore, it was demonstrated that the *E. coli* F18^+^ greatly affects the gut health and growth performance of pigs, whereas the novel synbiotic showed a potential to mitigate the effects of *E. coli* F18^+^ infection in an AGP-free diet.

## Data Availability Statement

The raw data supporting the conclusions of this article will be made available by the authors, without undue reservation.

## Ethics Statement

The experimental procedures used in this study were reviewed and approved by the Institutional Animal Care and Use Committee (IACUC) at North Carolina State University.

## Author Contributions

SK conceptualized the study and secured the funding. SK and JT designed the study. MD performed the experiments. MD and SK analyzed the data. The manuscript was written and reviewed by MD and SK. SK, MD, and JT discussed the results and approved the final manuscript. All authors contributed to the article and approved the submitted version.

## Conflict of Interest

JT was employed by the company BioResources International. The remaining authors declare that the research was conducted in the absence of any commercial or financial relationships that could be construed as a potential conflict of interest.
